# Mercury spikes as evidence of extended arc-volcanism around the Devonian–Carboniferous boundary in the South Tian Shan (southern Uzbekistan)

**DOI:** 10.1038/s41598-021-85043-6

**Published:** 2021-03-11

**Authors:** Michał Rakociński, Agnieszka Pisarzowska, Carlo Corradini, Katarzyna Narkiewicz, Zofia Dubicka, Nuriddin Abdiyev

**Affiliations:** 1 Institute of Earth Sciences, University of Silesia in Katowice, Faculty of Natural Sciences, Będzińska 60, 41-200 Sosnowiec, Poland; 2 Dipartimento di Matematica e Geoscienze, Università di Trieste, via Weiss 2, 34128 Trieste, Italy; 3 Polish Geological Institute, National Research Institute, Rakowiecka 4, 00-975 Warsaw, Poland; 4 University of Warsaw, Faculty of Geology, Żwirki i Wigury 93, 02-089 Warszawa, Poland; 5 Kitab State Geological Reserve, Shakhrisabz, Uzbekistan Kashkadarya Region

**Keywords:** Geochemistry, Environmental chemistry, Environmental impact, Palaeoceanography, Palaeoclimate, Climate sciences, Environmental sciences

## Abstract

Recently, the end-Devonian mass extinction (Hangenberg Crisis, 359 Ma) was identified as a first-order mass extinction, albeit not one of the “Big Five” events. Many marine and terrestrial organisms were affected by this crisis. The cause of this mass extinction is still conjectural and widely discussed. Here we report anomalously high mercury (Hg) concentrations from the South Tian Shan (Uzbekistan), together with correlation using conodont biostratigraphic data. Hg enrichment (to 5825 ppb) was detected in marine deposits encompassing the Hangenberg Crisis. In the Novchomok section, the Hangenberg Crisis interval does not contain typical Hangenberg Black Shales; however, by means of inorganic geochemistry (enrichment of redox-sensitive elements such as Mo, V, and U) we detected an equivalent level despite the lack of marked facies changes. This is the first record of Hg and Hg/total organic carbon anomalies in marly shales, marls and carbonates that are totally independent of facies changes, implying that volcanism was the most probable cause of the Hangenberg Crisis. This conclusion is confirmed by the presence of a negative δ^13^C excursion, which may reflect massive release of isotopically light carbon from volcanogenic and thermogenic devolatilization likely combined with increased arc-volcanism activity worldwide at the end of the Devonian.

## Introduction

The Hangenberg Crisis occurred at ca. 359 Ma and significantly affected both the pelagic realm (especially acritarchs, ammonoids, conodonts and many vertebrates)^1–3^ and benthic organisms such as trilobites and ostracods^4^. This extinction is linked to worldwide anoxia caused by global climatic changes^4–6^. The event also affected terrestrial ecosystems, causing the extinction of many fish and tetrapods as well as many land plants^3,4^. More than 50% of marine genera were lost during this extinction^4^.

The late Famennian interval was a time of intensive volcanic activity, which led to significant changes in the global climate and biosphere, and is widely thought to have caused the extinction event known as the Hangenberg Crisis^4–7^. Recently, the volcanic control on this biotic overturn was questioned by^8^, who postulated the influence of elevated UV-B radiation as a trigger for this event. These authors rejected volcanic trigger because they did not found Hg spikes in investigated terrestrial D–C boundary sections at east Greenland, which could be confirmed LIP activity^8^. However, recently large amounts of evidence for extensive volcanic activity expressed by Hg anomalies has been found in many parts of the world^6,7,9–12^ in different palaeogeographical and tectonic settings.

Volcanic eruptions and submarine hydrothermal activity are the main natural sources of mercury in recent and ancient environments, and are reflected by Hg spikes in sedimentary rocks^13–15^. Mercury is mainly emitted as gaseous Hg^0^, and has a long time of residence in the atmosphere (1–2 years); therefore this element is a very good tracer of ancient eruptions in contrast to many trace elements emitted by volcanoes, but with residence time of only several weeks (^16^ and references therein). For the first time, Sanei et al.^17^ used Hg as a proxy for volcanism at the Permian–Triassic boundary and attributed Hg outputs to the activity of the Siberian Traps. Over the past few years, Hg was successfully used as a reliable indicator of the connection between volcanic paroxysms and mass extinctions (e.g.,^7,15^).

Here we report for the first time very large anomalous Hg spikes from the south-western part of Tian Shan, together with correlation by means of conodont biostratigraphic data. Observed mercury anomalies in this area prove that intensive volcanic activity predated the Hangenberg Crisis, but also occurred during the event, providing key data to help resolve the controversy about the cause of this global event.

## Study area

**Figure 1:**
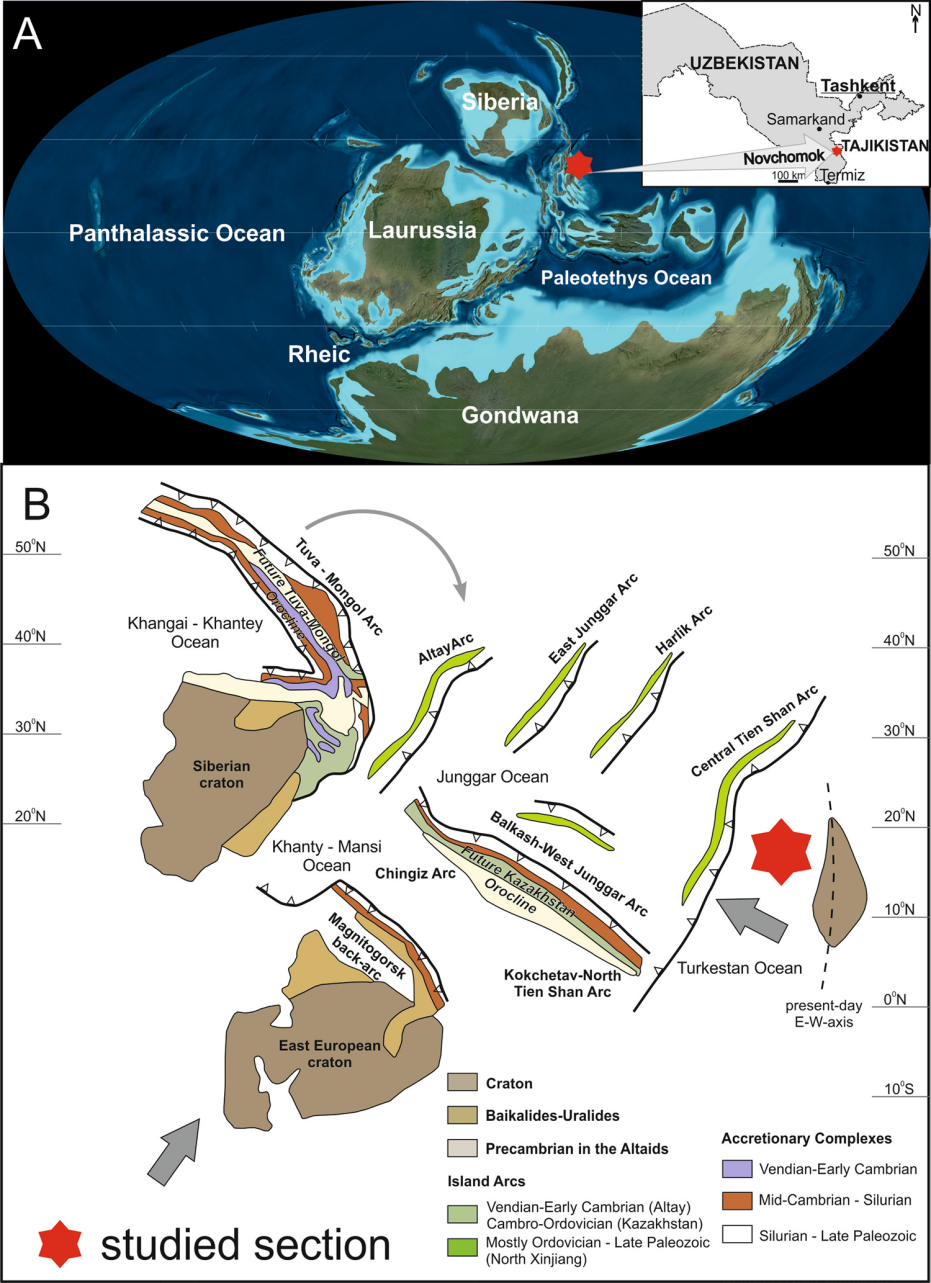
**(A)** Late Devonian (360 Ma) palaeogeography (after^91^ see also^12^) with the locations of Uzbekistan, the Novchomok section and the National Kitab Geological Reserve marked. (**B)** Tectonic model (after^78^) of the study area (shown by the red star), including an amalgamating cluster of island arcs and accretionary complexes (grey arrow indicate plate movements; for detail see Fig. 12 in^78^).

**Figure 2:**
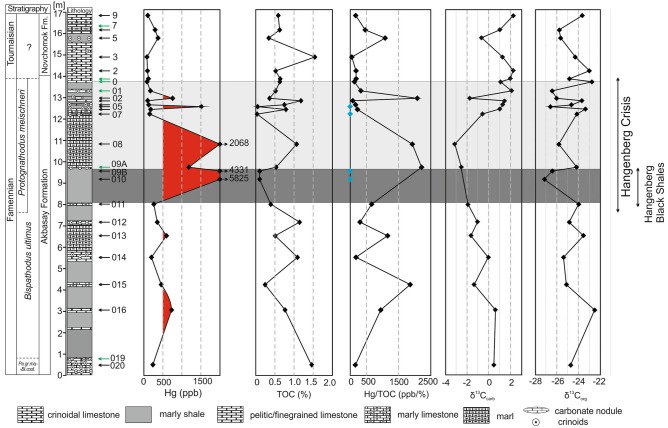
Composite plot of the Novchomok section showing the Hg (ppb) and TOC (%) contents, the Hg/TOC (ppb/%) ratio, and δ^13^C_org_ (‰) and δ^13^C_carb_ (‰). Samples with low TOC values (and therefore unreliable Hg/TOC values) are marked in blue. The red area is marked to indicate the mercury spikes (> 500 ppb Hg), in green are marked conodont samples.

**Figure 3:**
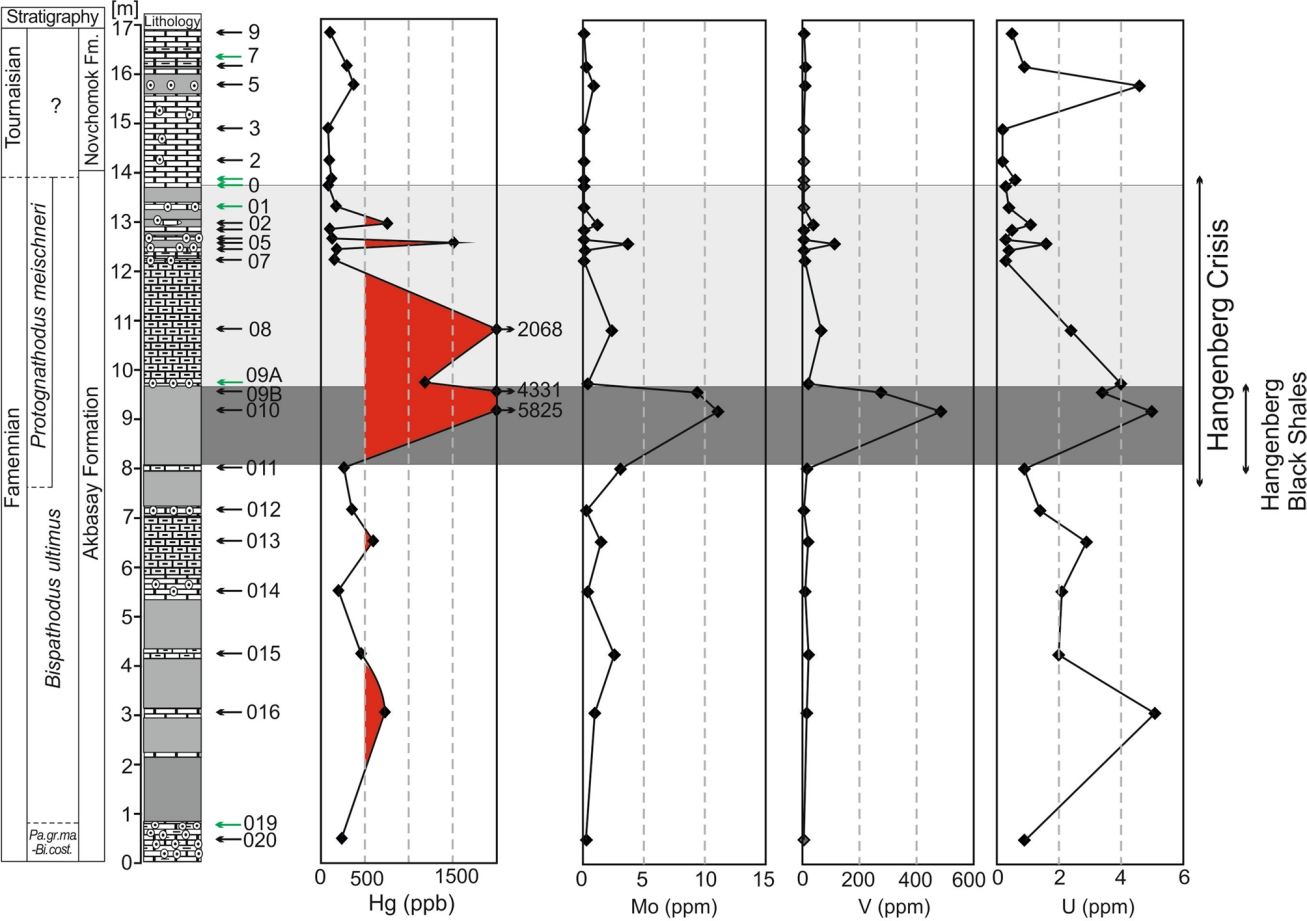
Composite plot of the Novchomok section showing the mercury, molybdenum, vanadium and uranium contents. See Fig. 2 for the explanation of the legend.

We examined a succession of deep-water, pelagic sedimentary rocks, encompassing the uppermost Devonian and Devonian–Carboniferous (D–C) boundary intervals (Fig. 1A) in the south-western part of Tian Shan, southern Uzbekistan. The Novchomok section is located on the right flank of the Dzhindy-Darya River in the eastern part of the Kitab State Geological Reserve, 170 km SSE of Samarkand, situated in the Zeravshan–Gissar Mountains area^18–21^. The sampled interval is ~ 17 m thick (Figs. 2 and 3), and comprises the top part of the Akbasay Formation and the base of the Novchomok Formation, which are characteristic of carbonate sediments of the Devonian–Carboniferous transition in this area^9,21^. The lower part of the investigated section (the Akbasay Formation) consists mostly of dark grey micritic limestones with crinoid detritus and corals, and also includes cherry-coloured and grey marly shales and marls. The upper part of the section (the Novchomok Formation) is composed of dark grey micritic limestones and brown marly shales, locally with crinoids. In the Novchomok section, the Hangenberg Crisis (HC) interval does not contain typical Hangenberg Black Shales (HBS); however, the anoxic interval was detected in the section by means of elevated levels of redox-sensitive elements, as described in the discussion.

The investigated area lies in the Zeravshan–Gissar mountainous area. The area belongs to the South Tian Shan, which is part of the eastern Central Asian Folded Belt^21^. Devonian and Carboniferous deposits were formed on the shelf of the Karakum–Tajik continent, within part of the passive margin of the Tarim plate, which consisted of Perigondwanan terranes located between the Palaeotethys and Asiatic oceans during this time^22–25^. The Novchomok section has been affected by strong thermal alteration: the maximum Conodont Alteration Index of the investigated rocks is 5, corresponding to temperatures of 300–480 °C^20^.

## Results

### Conodont biostratigraphy

Two zonation schemes are in use for the interval across the Devonian–Carboniferous boundary:^4^ and^26^ suggested a scheme similar to the previous one of Ziegler & Sandberg^27^, whereas^28^ and^29^ introduced a wide *Bi. ultimus* Zone that combines the former upper *expansa* and early and middle *praesulcata* zones.

**Figure 4:**
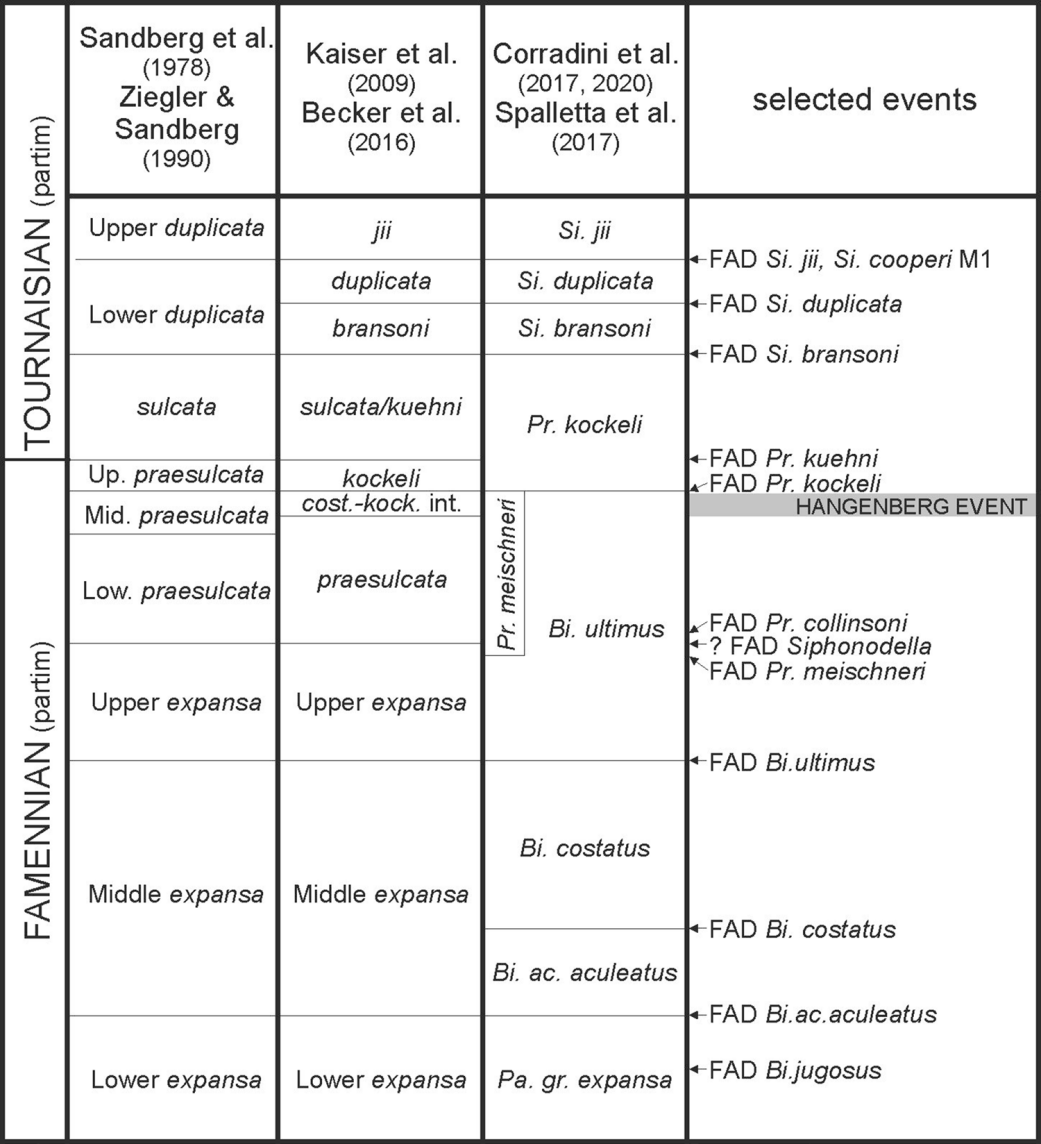
Conodont zonation applied in this paper (third column) compared with previous zonations and selected bioevents. Biostratigraphy is based on^26,28–31,92,93^. Thickness of biozones is calibrated according to their length, as estimated in the Devonian and Carboniferous chapter of *The Geologic Time Scale 2012*^94,95^. *Si.—Siphonodella, Bi.—Bispathodus, Pr.—Protognathodus, Pa.—Palmatolepis*.

Recently,^30^ proposed establishment of the *Protognathodus meischneri* Subzone for the upper part of the *Bi*. *ultimus* Zone (see^31^: this subzone is approximately equivalent to the Lower *praesulcata* Zone of Ziegler & Sandberg^27^ and to the *praesulcata* Zone of^26^ (Fig. 4). In the absence of the nominal species, the characteristic taxa are *Siphonodella praesulcata* Sandberg and *Protognathodus collinsoni*, the first occurrences of which are slightly above the first appearance datum (FAD) of *Pr*. *meischneri*^28^.

As no *Siphonodella* representatives were found in the studied Novchomok section, the zonation of^29^ was applied in the present study, including the new *Pr. meischneri* Subzone. However, because the index taxa are missing, the present biostratigraphy is based on compilation of the stratigraphic ranges of other taxa found in particular samples, using the data of^29^. Moreover, it should be stressed that scarcity and poor preservation state of conodonts have a negative effect on the precision of the biostratigraphic calibration.

**Figure 5:**
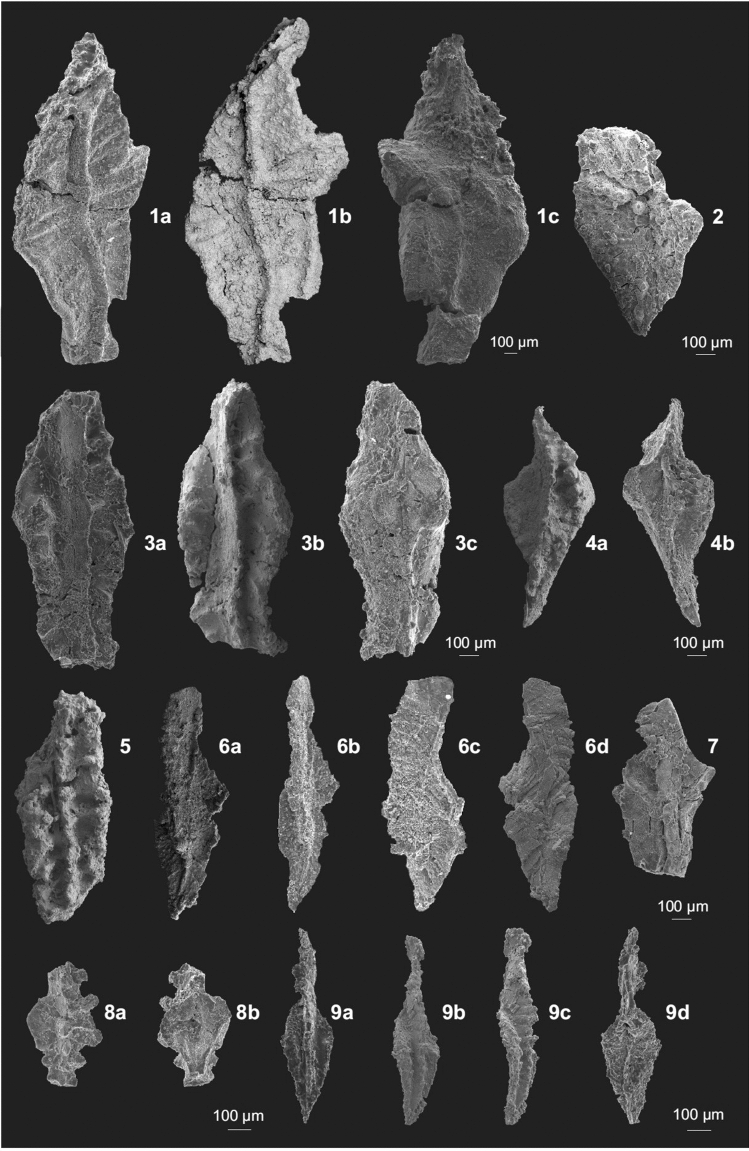
Conodonts from the Novchomok Section 1. *Pseudopolygnathus* sp. (1a. oblique, 1b. upper, 1c. lower views, sample 019, MUZ PIG 1825.II.11), 2. *Polygnathus* aff. *Po*. *styriacus* Ziegler (upper view, sample 09A, MUZ PIG 1825.II.12), 3. *Pseudopolygnathus* sp. (3a. upper, 3b. oblique, 3c. lower views, sample 019, MUZ PIG 1825.II.13), 4. *Pseudopolygnathus controversus* Morphotype 1 Sandberg and Ziegler (4a. upper, 4b. lower views, sample 019, MUZ PIG 1825.II.1), 5. *Pseudopolygnathus* aff. *Ps*. *primus* Branson and Mehl (5. upper view, sample 019, MUZ PIG 1825.II.14), 6. *Palmatolepis* sp. (6a. upper/oblique, 6b. upper, 6c. lateral, 6d. lower views, sample 09A, MUZ PIG 1825.II.15), 7. *Polygnathus vogesi* Ziegler (7. upper view, sample 01, MUZ PIG 1825.II.16), 8. *Bispathodus* cf. *B*. *aculeatus aculeatus* (Branson and Mehl) (8a. upper, 8b. lower views, sample 09A, MUZ PIG 1825.II. 2), 9. *Polygnathus communis dentatus* Druce (9a. upper, 9b. oblique, 9c. lateral, 9d. lower views, sample 019, MUZ PIG 1825.II.17).

**Figure 6:**
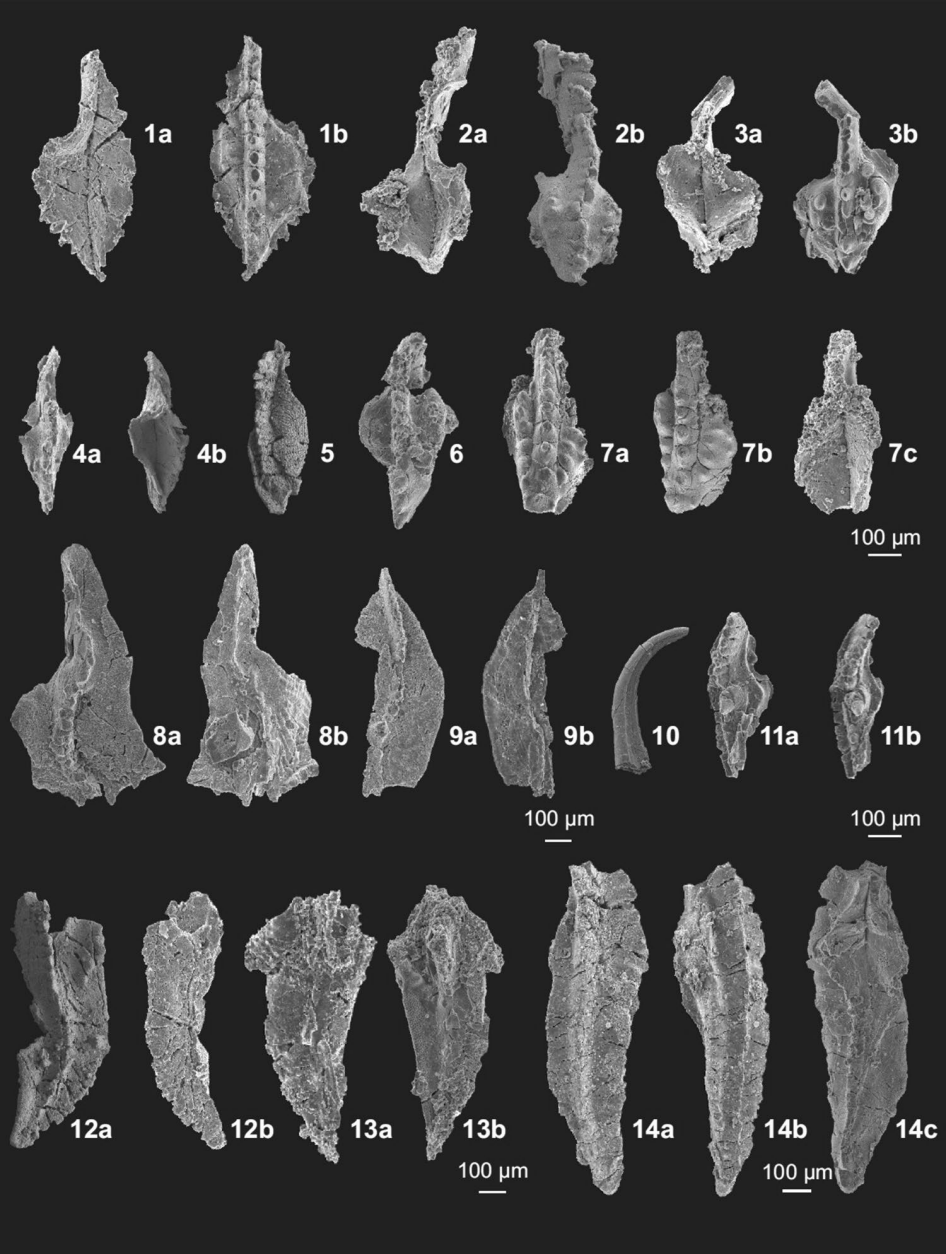
Conodonts from the Novchomok section. Most conodont elements illustrated in this figure are from sample Nov. 22-0, except the specimens in parts 9 and 14. 1, 2, 4. *Protognathodus collinsoni* Ziegler (1a. lower, 1b. upper views, MUZ PIG 1825.II.3; 2a. lower, 2b. upper views, MUZ PIG 1825.II.4; 4a. upper, 4b. lower views of juvenile form, MUZ PIG 1825.II.18), 3. *Protognathodus collinsoni* Ziegler → *Protognathodus kockeli* (Bischoff) (3a. lower, 3b. upper views, MUZ PIG 1825.II.5), 5, 9. *Palmatolepis* sp. (5. upper view of juvenile form, sample Nov. 22–0, MUZ PIG 1825.II.9; 9a. upper, 9b. lower views, sample Nov. 22–1, MUZ PIG 1825.II.10), 6. *Pseudopolygnathus* cf. *Ps*. *brevipennatus* Ziegler (6. upper view, MUZ PIG 1825.II.6), 7.*Pseudopolygnathus* aff. *Ps*. *primus? *Branson and Mehl (7a. upper, 7b. upper/oblique, 7c. lower views, MUZ PIG 1825.II.7), 8. *Palmatolepis perlobata* Ulrich and Bassler (8a. upper, 8b. lower views, MUZ PIG 1825.II.8), 10. *Panderodus* sp. (10. obverse view, MUZ PIG 1825.II.19), 11. *Palmatolepis gracilis sigmoidalis* Ziegler (11a. oblique, 11b. upper views, MUZ PIG 1825.II.20), 12, 13. *Polygnathus* sp. (12a. upper, 12b. lower/oblique views, MUZ PIG 1825.II.21; 13a. lower, 13b. upper views, MUZ PIG 1825.II.22), 14. *Pseudopolygnathus* sp. (14a. upper, 14b. lower, 14c. oblique views, sample Nov. 22–7, MUZ PIG 1825.II.23).

The age of the lowermost sample (Nov 22-019) is constrained by the total stratigraphic range of *Pseudopolygnathus controversus* Sandberg and Ziegler (Fig. 5: 4) to the interval from the upper part of the *Palmatolepis gracilis manca* Zone to the *Bispathodus costatus* Zone. If the occurrence of *Bispathodus* cf. *aculeatus aculeatus* (Branson and Mehl) (Fig. 5: 8), which appears higher in the section in sample Nov 22-09A, corresponds to the FAD of this subspecies, it would constrain the age of sample Nov 22-019 to the interval of the *Pa. gr. manca* to *Pa. gr. expansa* zones. The low abundance of conodonts in the discussed samples, however, makes such age determination risky. The age of assemblages from samples Nov 22-09A and Nov 22-01 is estimated as the interval from the *Bispathodus ac*. *aculeatus* Zone to the *Bispathodus ultimus* Zone. The upper limit is set by the first appearance in the section, in sample Nov 22-0, of *Protognathodus collinsoni* (Fig. 6: 1, 2).

The richest sample Nov 22-0 is attributed to the *Pr*. *meischneri* Subzone within the upper part of the *Bispathodus ultimus* Zone. This age assignment is based on the occurrence of *Pr*. *collinsoni* specimens (Fig. 5: 1, 2) and a lack of typical representatives of the latter species. More precisely, the occurrence of a transitional element between *Pr*. *collinsoni* and *Pr*. *kockeli* (Fig. 5: 3) may suggest the upper part of the *Pr. meischneri* Subzone. In the same sample, species not previously reported at such a high stratigraphic level occur: *Pseudopolygnathus* cf. *brevipennatus* (Fig. 5: 6, 7) and *Palmatolepis perlobata* ssp. (Fig. 5: 8). These taxa became extinct in the lower part of the *Bi*. *ultimus* Zone^29^. Their occurrence may be explained by reworking from older strata, as suggested also by other fragments of *Palmatolepis* (Fig. 5: 5 and Fig. 5: 9—from the sample Nov 22-1) that recall older forms. However, with the exception of the scarce reworked elements, the remaining assemblage fits well in this interval, as no younger species are present. The fact that there are no species which have their FAD in the *Pr. kockeli* Zone (*Pr*. *kockeli*, *Polygnathus purus subplanus*, *Po. politus*) strongly supports our attribution. Reworking of older conodonts is a common phenomenon associated with the Devonian-Carboniferous boundary^4^.

In summary, the investigated strata of the Novchomok section, spanning the interval between samples Nov 22-019 and Nov 22-0, certainly belong to the uppermost Famennian. It should be stressed, however, that because of the scarce biostratigraphic data the succession of biozones (SD. 1) is hard to define precisely. The key sample Nov 22-0 gives the most precise age constraint, i.e. the upper part of the *Protognathodus meischneri* Subzone within the *Bi. ultimus* Zone. In turn, the assemblage from the sample Nov 22-019 is certainly older than the *Bi. ultimus* Zone, as explained above. Thus, the interval between samples Nov 22-019 and Nov 22-0 most probably includes the *Bi. ultimus* Zone. Sample Nov 22-0 is located very close to the D–C boundary, and sample Nov 22-1 is probably also very close to the boundary.

## Carbonate and organic carbon isotopes

The δ^13^C_carb_ values from the Novchomok section gradually decrease from 0‰ to − 3.0‰ from the *Bi. ac. aculeatus* Zone (samples Nov 22-020 to Nov 22-012) into the *Pr. meischneri* Subzone (samples Nov 22-011 to Nov 22-08; near the equivalent of the Hangenberg Black Shale; see below). A distinct positive excursion of 5‰ was detected in the interval including the top of the Akbasay Formation and the base of the Novchomok Formation. The negative δ^13^C_org_ shift of 3‰ from the lower part (samples Nov 22-020 to Nov 22-011) to middle part (samples Nov 22-010 to Nov 22-09B) of the Akbasay Fm corresponds to time-equivalents of the HBS (see below). A less pronounced positive δ^13^C_org_ excursion (to -23‰) parallels the δ^13^C_carb_ excursion in the D–C boundary interval (Fig. 2). The negative δ^13^C excursion corresponds to the largest Hg peaks (see below; SD. 3).

Carbon isotopes in marine carbonates reflect a real change in ocean chemistry but maybe also affected by diagenetic alteration. Diagenetic processes are particularly evident for negative carbon isotope shifts. The isotopic composition of bulk rock organic matter is influenced by variable sources of input and differential degradation of organic components^32^. An increase of thermal maturation is typically associated with ^13^C enrichment^33^. Therefore, the higher δ^13^C_org_ measured in the TOC-poor samples may reflect the thermally induced mobilization compounds that are enriched in ^12^C relative to the bulk of the organic carbon (^33,34^; SD 3). Lower δ^13^C values within the HBS equivalent (see below) may be explained either by incorporation of ^12^C derived from oxidized organic matter (δ^13^C_carb_), a change in the TOC composition (δ^13^C_org_)^35^, or liberation of massive amounts of ^13^C-depleted CH_4_ and CO_2_ e.g.^36^. The latter were suggested as a major contributors to the negative excursion of both carbonate and organic carbon isotopes during e.g. the Permian–Triassic boundary transition e.g.^37^ and Early Toarcian^38^. A comparison of δ^13^C variation curves in the Novchomok and the other D/C sections from different facies of distant continents reveals similar variation patterns (see below), which may be explained by changes in the global carbon cycle.

## Redox-sensitive trace elements and total organic carbon contents

Redox conditions can be deduced on the basis of indices such as the Th/U and C_org/_P ratios^39–41^, as well as from enrichment of redox-sensitive trace metals such as Mo, U and V^42,43^. The Th/U ratio of anoxic siltstones or shales is less than 3, whereas in carbonates this ratio is typically below 1 (for details see references^39,40^). The C_org_/P ratio in anoxic conditions is greater than 150, whereas for sediments formed under oxic conditions the value is below 30. Intermediate values are characteristic of dysoxic or high-productivity and periodically oxygen-restricted settings^41^.

In the studied section, the molybdenum values in many samples (especially in the upper part of the section) were very low (< 0.5 ppm, often below the detection limit of 0.1 ppm; Table 1). In contrast, in samples enriched in Hg, Mo levels were generally > 1 ppm with a maximum value of 11.1 ppm. Uranium levels in the samples were between 0.2 to 5.1 ppm. The Th/U ratios in samples enriched in Hg were low, < 1 in carbonates and < 3 in shales, indicating oxygen-depleted benthic redox conditions. However, these low values could in some horizons be an artifact associated with strong depletion in thorium, as well as other detrital-fraction elements. Interestingly, total sulphur was below the detection limit (< 0.02%) in the entire section. The low C_org_/P ratios throughout the section are characteristic of low-productivity and oxic conditions, which is confirmed by the very low total organic carbon (TOC) values of less than 0.2%. Horizons with Hg anomalies are characterized by higher V values of 17–486 ppm (Fig. 3; Table 1), whereas in almost all samples from other parts of the section the V content is below the detection limit, implying more oxic conditions. The level equivalent to HBS is also enriched in uranium and molybdenum (Fig. 3): even if the concentrations of the elements have been depleted by diagenesis, they still indicate more restricted conditions.

**Table 1:** Hg (ppb), TOC (%), Hg/TOC (ppb/%) ratio, CaCO_3_, Al_2_O_3_, Fe_2_O_3_, total sulphur (TS, %), Mo (ppm), As (ppm), V (ppm), U (ppm), Th (ppm), P (%), Th/U and C_org/_P ratios, δ^13^C_org_ (‰) and δ^13^C_carb_  (‰). Abbreviation: ls – limestone, m.sh. – marly shale, sh. – shale.

Fm	Sample	Lith	Hg (ppb)	TOC (%)	Hg/TOC (ppb/%)	CaCO_3_ (%)	Al_2_O_3_ (%)	Fe_2_O_3_ (%)	TS (%)	Mo (ppm)	As (ppm)	V (ppm)	U (ppm)	Th (ppm)	P (%)	Th/U	C_org_/P	δ^13^C_org_ (‰)	δ^13^C_carb_ (‰)
Novchomok	NOV 22 /9	ls	102.64	0.59	173.66	88.52	0.07	0.09	0.0011	< 0.1	0.6	8	0.5	< 0.2	0.02	n.d	27.09	− 23.7	2.3
	NOV 22 /6	m.sh	294.74	0.63	468.25	39.35	2.03	0.49	0.0000	0.3	7.0	12	0.9	2.6	0.02	2.89	36.06	− 25.8	1.0
	NOV 22 /5	m.sh	373.30	0.34	1085.87	23.33	7.86	1.44	0.0000	0.9	42.9	11	4.6	8.8	0.02	1.91	15.76	− 25.7	− 0.7
	NOV 22 /3	ls	80.38	1.55	51.95	85.81	0.07	0.06	0.0013	< 0.1	< 0.5	< 8	0.2	< 0.2	0.04	n.d	39.39	− 24.3	1.3
	NOV 22 /2	ls	94.59	0.52	181.65	95.62	0.11	< 0.04	0.0016	< 0.1	< 0.5	< 8	0.2	< 0.2	0.04	n.d	11.93	− 23.0	2.3
Akbasay	NOV 22 /1	ls	119.95	0.64	187.30	87.39	0.44	0.14	0.0000	< 0.1	< 0.5	< 8	0.6	0.4	0.24	0.67	2.62	− 24.8	2.0
	NOV 22 /0	ls	83.68	0.63	132.44	81.46	0.12	0.10	0.0010	< 0.1	< 0.5	< 8	0.3	< 0.2	0.09	n.d	6.89	− 22.8	1.1
	NOV 22 /01	ls	173.44	0.52	332.92	94.37	0.08	0.09	0.0000	< 0.1	< 0.5	< 8	0.4	< 0.2	0.09	n.d	5.97	− 26.5	2.1
	NOV 22 /02	sh	756.22	0.36	2086.64	8.98	2.98	1.39	0.0000	1.2	20.8	40	1.1	3.0	0.30	2.73	1.20	− 26.0	− 1.7
	NOV 22 /03	ls	100.94	1.18	85.26	88.61	0.1	0.14	0.0004	< 0.1	1.0	< 8	0.5	< 0.2	0.08	n.d	14.28	− 23.7	1.4
	NOV 22 /04	ls	127.86	0.75	170.59	88.85	0.02	0.15	0.0004	< 0.1	1.2	< 8	0.3	< 0.2	0.05	n.d	15.61	− 24.7	1.3
	NOV 22 /05	sh	1512.52	0.05*	28,784.13*	0.61	7.51	3.25	0.0013	3.7	49.2	114	1.6	5.5	0.20	3.44	0.26	− 26.6	b.d
	NOV 22 /06	ls	179.95	0.79	227.50	80.17	0.11	0.22	0.0000	0.2	2.6	< 8	0.4	< 0.2	0.15	n.d	5.18	− 23.4	1.0
	NOV 22 /07	ls	151.85	0.04*	3973.71*	89.00	0.16	0.17	0.0009	< 0.1	3.5	10	0.3	< 0.2	0.09	n.d	0.42	− 24.2	− 0.6
	NOV 22 /08	m.sh	2068.39	1.07	1929.19	46.12	1.04	0.46	0.0001	2.4	9.9	67	2.4	1.2	0.03	0.50	35.10	− 25.8	− 3.1
	NOV 22 /09A	ls	1186.64	0.53	2219.75	86.76	0.18	0.12	0.0000	0.4	1.9	22	4	0.3	0.03	0.08	20.42	− 24.2	− 2.5
	NOV 22 /09B	sh	4331.47	0.10*	42,057.13*	1.07	3.76	1.63	0.0022	9.4	40.6	276	3.4	2.3	0.05	0.68	2.15	− 26.4	b.d
	NOV 22 /010	sh	5825.32	0.10*	59,159.06*	0.27	4.94	1.48	0.0002	11.1	37.7	486	5	4.0	0.01	0.80	11.28	− 27.2	b.d
	NOV 22 /011	ls	263.48	0.39	667.62	74.16	0.99	0.65	0.0034	3.1	9.6	18	0.9	0.8	0.06	0.89	6.46	− 24.0	− 1.9
	NOV 22 /012	ls	352.93	1.15	307.45	84.82	0.25	0.21	0.0001	0.3	2.3	< 8	1.4	0.4	0.09	0.29	12.53	− 24.9	− 1.0
	NOV 22 /013	m	594.98	0.51	1165.86	66.43	1.07	0.49	0.0018	1.5	8.2	21	2.9	0.8	0.09	0.28	5.57	− 23.6	− 1.6
	NOV 22 /014	ls	200.13	1.09	182.95	88.73	0.43	0.11	0.0000	0.4	2.1	11	2.1	0.4	0.07	0.19	16.71	− 25.4	0.0
	NOV 22 /015	ls	457.68	0.25	1862.11	74.60	1.38	0.53	0.0004	2.6	12.5	23	2	0.9	0.07	0.45	3.75	− 25.1	− 1.3
	NOV 22 /016	ls	730.27	0.77	949.28	84.92	0.44	0.23	0.0010	1.0	2.8	17	5.1	0.5	0.03	0.10	22.04	− 22.5	0.6
	NOV 22 /020	ls	238.74	1.47	162.89	75.61	0.93	0.17	0.0000	0.3	2.1	< 8	0.9	1.1	0.06	1.22	23.99	− 24.7	0.5

## Mercury spikes around the Devonian–Carboniferous boundary

Hg enrichment was observed in several horizons in the Akbasay Formation (uppermost Famennian) in the carbonate-rich, organic-poor Novchomok section (Fig. 2), where TOC values were 0.1% to 1.07% in the interval correlated with the Hangenberg Black Shale. The Hg contents in the Novchomok Formation are 80–373 ppb, similar to the background Hg values of 84–352 ppb in the Akbasay Formation. However, the Akbasay Formation yielded anomalous Hg contents of 457–5825 ppb in some horizons (Table 1); in contrast, the average Phanerozoic Hg abundance varies from 30 ppb in limestone to 450 ppb in argillaceous shale^44^. In general, Hg is enriched to different extents in all limestone samples (Table 1). The Akbasay Formation is characterized by some intervals with very high Hg/TOC values, from 949 to as much as 2220 ppb/%, which are ~ 4.5–11 times higher than the value of ~ 200 ppb/% of the background deposits.

The crucial horizons with anomalous Hg values are a dark gray marly shale, limestone and marls (Figs. 2 and 3). An abrupt increase in Hg concentrations and the largest Hg/TOC peak coincide with the negative excursion in carbonate and organic δ^13^C (Fig. 2, SD. 3).

## Discussion and conclusions

The Hangenberg Crisis is associated with a transgression pulse, development of anoxic conditions in the sea, and climate warming e.g.^4–6,12^. Several possible primary causes of the Hangenberg Crisis have been proposed: climate and glacioeustatic changes; global carbonate crisis resulting from oceanic acidification; salinity changes; phytoplankton blooms and expansion of land plants; volcanism; and extraterrestrial impact (e.g.^4,6,45^. There is as yet no clear consensus about the cause of the event, and the primary triggers are still a matter of vigorous debate.

In the Novchomok section the HC interval does not contain typical HBS. The anoxic interval was detected in the studied section, however, by means of elevated Mo concentrations (Fig. 3, Table 1) in the dark shaly–marly package (Fig. 2), which may be regarded as the stratigraphic equivalent of the HBS horizon. The occurrence of the HBS equivalent implies that the base of the *meischneri* Subzone is located somewhere below the dark interval, i.e. below sample Nov 22-011. A similar late first occurrence of species of the genus *Protognathodus* has been documented in other localities and has been explained by ecological factors^46^.

Submarine hydrothermal activity and volcanic eruptions are the main natural sources of mercury in recent and ancient environments, and are reflected by Hg spikes in sedimentary rocks^13–15^. Interestingly, all the “Big Five” mass extinctions are associated with mercury spikes, supporting extensive volcanism having been a primary cause of environmental changes during these events^7,17,47–51^.

Recently, the volcanic “smoking gun” as a potential trigger of the Hangenberg Crisis has been questioned^8^. According to^8^, elevated UV-B radiation and a drastic drop in stratospheric ozone during global warming were responsible for this biotic overturn. Moreover, Fields et al.^52^, postulated supernova explosions as a trigger for the event. These hypotheses are very attractive, but there is a lack of hard evidence to support the extraterrestrial scenario, whereas the occurrence of extensive volcanic activity during that time in many parts of the world confirms an Earth-bound scenario (e.g.^6,7^).

**Figure 7:**
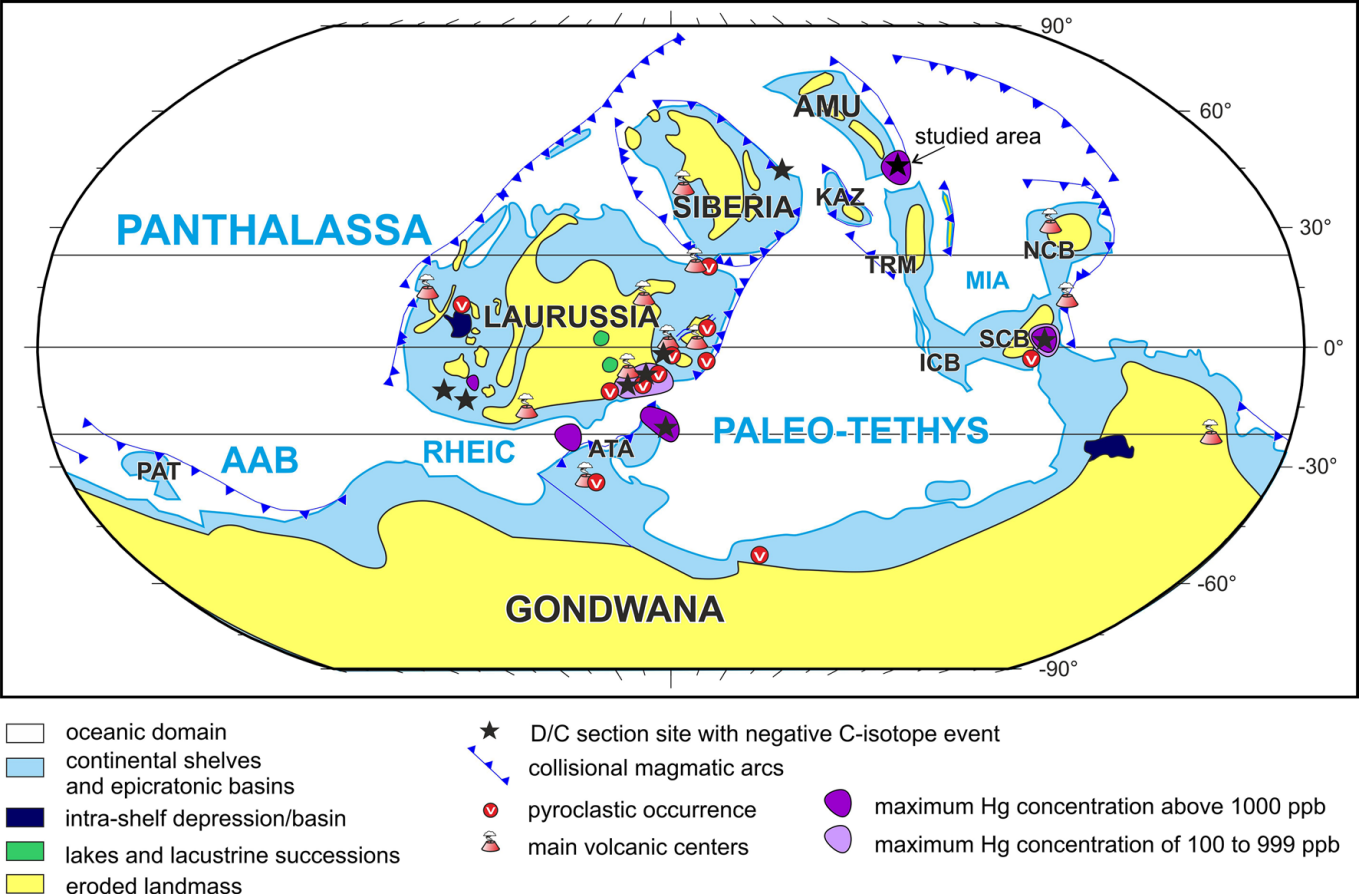
Distribution map of the latest Famennian magmatic activity centers, and the location of pyroclastic and mercury-enriched deposits. Palaeogeographic map modified after [Fig. 14 in^6^: https://doi.org/10.1016/j.gloplacha.2020.103155; see also^96^). Hg concentrations based on this study and after^6,97^. The most likely candidate for the observed Hg anomalous contents is arc magmatism. *AAB* Arequipa–Antofalla Ocean, *AMU* Amuria, *ATA* Armorican Terrane Assemblage, *ICB* Indochina, *KAZ* Kazakhstan, *MIA* Mianlue Ocean, *NCB* North China, *PAT* Patagonia, *SCB* South China, *TRM* Tarim.

In many areas pyroclastic horizons occur in the uppermost Famennian (Fig. 7), such as in Poland^5,6,53^, Germany (^7,54–56^), the Iberian Peninsula^57^ and China^58^. Recently, convincing evidence of increased volcanic and hydrothermal activity as given by mercury spikes has been detected in the HC interval in Vietnam^10^; the Czech Republic^11^; south China^7,11^; the Holy Cross Mountains, Poland^7^; Thuringia and Bavaria, Germany^6,7,9^; and the Carnic Alps in Austria and Italy^6,12^. Menor-Salvan et al.^57^ described a hydrothermal peak in the Iberian Peninsula, the last phase of which (at ~ 360 Ma) formed the world’s largest cinnabar ore reservoir in Almadén.

Previously documented mercury spikes^9^ in the Novchomok section, which is now dated by means of conodonts, partly predate the biocrisis; however, the main and upper Hg spikes encompass the HC interval (Fig. 2), confirming the volcanic scenario for this event. Our results from Novchomok include five Hg anomalies, of which the two lower anomalies (samples Nov 22-016 and Nov 22-013) predate the Hangenberg Crisis (Fig. 2), whereas the upper spikes (samples Nov 22-010 to Nov 22-05) could be related to the end-Devonian event.

The lower part of the Hangenberg Crisis interval (sensu^4^) is characterized by a globally observed δ^13^C negative excursion in the carbonate^59–62^ and organic-matter records^6^. The minima in the carbon isotope curves coincide with Hg enrichment and an Hg/TOC excursion in surprisingly many places around the world in the initial phase of the HC^64^. According to some authors^64^, Hg enrichment is connected to occurrences of Hg-enriched sulphides and cannot be interpreted as a volcanic proxy, especially for the Ordovician–Silurian, Frasnian–Famennian and Permian–Triassic boundaries. According Shen et al.^65^, a sulfide host phase for Hg occur in strongly euxinic environments with high TS contents (> 1.0%). Still, generally, Hg is most commonly associated with the organic fraction. However, other authors^43^ have proposed that neither Hg/TOC nor Hg/S are significantly linked with the organic and sulphide fractions, and therefore are useful as a volcanic proxy (compare^66^). These ratios are not significantly influenced by redox conditions^7,43^. This absence of linkage can be confirmed in our investigated section, because the observed spikes occur independently of lithology, in limestones and marls as well as in shales. The Hg vs. TOC correlation in the Novchomok section is very low (R^2^ = 0.16; SD. 3), indicative of a volcanic Hg source, not a bioproductivity-controlled Hg oversupply^7^. The Hg vs. Al_2_O_3_ correlation in the investigated successions is also very weak (R^2^ = 0.24), indicating no correlation of Hg with the clay fraction and terrigenous input. In addition, the entire investigated section is strongly impoverished in total sulphur (trace amounts, Table 1), which excludes the sulphide fraction as a host of mercury.

Negative δ^13^C excursions can reflect massive release of isotopically light carbon from volcanogenic and thermogenic devolatilization in a giant volcanic system (^67^; see R&S hypothesis in^63^). The killing effectiveness of a volcanic cataclysm depends on the geological setting of the host region, the size of the igneous province, the magma plumbing system and the eruption dynamics^68^. The form and eruption dynamics of the volcanic system control the magnitude and composition of the thermogenic outgassing that most probably causes the greatest disruption to the carbon cycle (e.g.^69–71^). Moreover, a large igneous province (and possibly arc magmatism and arc–continent collisions) can drive anoxia via global warming^68,72^; therefore, the global D–C biodiversity crisis, similarly to the Permian–Triassic extinction^73^, may have been mainly driven by volcanism-linked anoxia. As described by^12^, the presence of 55 pg/g dry weight of MeHg in the HBS interval (sample Nov 22–010) could indicate methylmercury poisoning, which may have been an additional driver of the end-Devonian Mass Extinction (for detail see^12^).

One of the problems with the volcanism hypothesis is a lack of precisely dated Large Igneous Provinces (LIP) during this time interval (^74^, for details see^7^). Nevertheless, the activity of the continental silicic Maritimes (Magdalene) LIP in eastern Canada includes the 360–370 Ma interval, with a pulse at around 360 Ma^75,76^. Moreover, LIPs often precede the main extinction intervals, which can be explained by delayed ecosystem responses, because volcanism leads to climatic changes (warming)^7^ (the press–pulse volcanic model;^7^). According to Ernst et al.^76^, a second main pulse of the Kola–Dnieper and Yakutsk–Vilyui LIPs occurred around 360 Ma, which may be tentatively correlated with previous Famennian ocean anoxic episodes such as the *Annulata* or Dasberg Events and/or the Hangenberg Crisis.

Another possibility is that intraplate (?oceanic;^4^) LIPs were consumed in subduction zones; in this case, Hg enrichment could be the only reliable evidence for increased volcanic activity at the planetary scale^7^. Many magmatic and volcanic rocks (Fig. 7) as well as ash layers and hydrothermal deposits occur near the D–C boundary, associated with observed mercury enrichments in different palaeogeographic regions^6,7^, confirming that the Late Devonian was a time of many active magmatic centres.

The high mercury concentration in the Novchomok section may also suggest an especially close location to a volcanic Hg source, which may have been submarine volcanic activity associated with hydrothermal pulses, see e.g.^6^ and^77^. The investigated area of the Tarim plate lay close to the active Central Asian Orogenic Belt during the Late Devonian and early Carboniferous^78^ and the arc-volcanic Magnitogorsk zone (Fig. 1B). The zircon ages of this zone include the D–C boundary time^79,80^, and so the zone could potentially have been the source area of Hg.

However, the main activity of the Devonian magmatic centre in Magnitogorsk Zone predated the Hangenberg Crisis. According to^81^, the Magnitogorsk island arc was active only until the end-Frasnian; if this is the case, another Hg source must be sought. In fact, volcanic activity appears to have been waning at the end of the Devonian Period in the east Magnitogorsk zone, but was still occurring^82^. Additionally, dating of sulphide mineralisation in the Urals volcanic-hosted massive sulphide deposits (362 ± 9 and 363 ± 1 Ma;^80^) and intrusive magmatism in the Ural Platinum Belt, where continental-margin gabbro–tonalite–granodiorite magmatism was initiated at ~ 365–355 Ma^83,84^, include the time interval of the end-Devonian crisis. Many pyroclastic rocks related to volcanism within an intra-oceanic arc occur around the D–C boundary at Baruunhuurai Terrane, Mongolia^85^; however, precise biostratigraphic data are not available for this interval. It is also possible that the Hg-source island arc was consumed in a subduction zone during closure of the Uralian or Turkestan Ocean.

Marshall et al.^8^ described floral malformations in east Greenland and interpreted the observed floral mutagenesis to be a result of elevated UV-B radiation, reflecting ozone-layer reduction that was suggested to have been associated with global warming. Surprisingly, according to those authors, mercury data excluded planetary-scale volcanism as a potential trigger for the end-Devonian Hangenberg Crisis.

However, according to another study^86^, floral malformations during this time interval were associated with the mutagenic effect of regional acidification caused by explosive (arc-type?) volcanism, recorded in common pyroclastic horizons. Visscher et al.^87^ and Foster & Afonin^88^ argued that this type of floral mutagenesis observed at the Permian–Triassic boundary reflected the biotic response to environmental stress associated with increased volcanic activity of the Siberian Traps coupled with degradation of the ozone layer and increased UV radiation. According to^89^, volcanic activity of the Central Atlantic Magmatic Province and SO_2_ emissions were responsible for malformation of plant cuticles. Thus, plant mutagenesis supports, rather than excludes, a volcanic scenario. LIP-related cooling connected to darkness and occurrence of SO_2_ and its products in the atmosphere is rather short-term, in contrast to global warming induced by release of volcanogenic CO_2_, which could have caused ozone damage and a consequent increase in UV-B radiation^90^. Furthermore, as shown in the study by^77^ for the Palaeocene–Eocene Thermal Maximum, the occurrence of Hg and Hg/TOC anomalies may be related to phreatomagmatic eruptions and submarine degassing from hydrothermal vent complexes leading to local deposition of Hg-enriched sediments.

The predicted causal relationship between large-scale volcanic activity, volcanically driven climatic and redox changes, and the response of the global carbon cycle is clearly visible in the end-Devonian (and also, for example, the end-Frasnian, P–T boundary and end-Triassic) stratigraphic (and biotic) record(s). In contrast, there is no hard evidence of an extraterrestrial cause of the Hangenberg Crisis, such as a supernova explosion.

## Methods

### Micropalaeontological preparation

Recovery of conodont elements was attempted from nine samples from the presumed D–C boundary interval, comprising the boundary between the Akbasay and Novchomok formations (SD. 1). Sample processing was carried out in the micropalaeontological laboratory of the Polish Geological Institute – National Research Institute, in Warsaw. The rock material—mostly dark grey, poorly metamorphosed limestones—was dissolved in 20% formic acid, and the insoluble residuum was enriched using heavy liquid (sodium metatungstate). No identifiable conodont elements were found in samples Nov 22-015, Nov 22-07 and Nov 22-3. All of the conodont elements are stored in the Polish Geological Institute—National Research Institute, in Warsaw.

In general, the obtained microfossils were difficult to identify because of their poor preservation. Much of the material consists of conodont fragments that are difficult to determine even at the genus level. The elements that were selected for taxonomic description are broken, fractured, twisted, compacted, plastically deformed and commonly covered with sediment and with authigenic mineral overgrowths. The high temperatures (300–480 °C) indicated by the Conodont Alteration Index values (see “Study area”) may indicate the occurrence of regional heating associated with deep burial and/or weak metamorphism. Tectonic stress caused fracturing of the specimens, leading to their disintegration during sample processing.

The frequency of taxa in the samples is listed in Supplementary Materials (SD. 1). The total number of tabulated specimens is 125, most of which (81%) came from sample Nov 22-0. Identified conodonts that are important for biostratigraphy are illustrated in Figs. 5 and 6; other characteristic specimens are figured in the Supplementary Materials (SD. 2).

### Isotope analysis

Bulk-rock samples for sedimentary organic carbon isotope analysis were pulverized and acidified with excess 10% HCl and held at 60 °C for at least 8 h to remove inorganic carbonate material. Samples were then rinsed with ultrapure (> 18 MΩ) deionized water to remove acid and oven-dried at 60 °C. Analyses of the isotope signature of organic carbon in sediment (δ^13^C_org_) were performed using a Thermo Flash EA 1112HT elemental analyser combined with a Thermo Delta V Advantage isotope ratio mass spectrometer in continuous-flow mode at the Institute of Geological Sciences, Polish Academy of Sciences (Warsaw). Isotope values for carbon are given in parts per thousand (‰) relative to the Vienna PeeDee Belemnite (VPDB) standard and calibrated according to certified international standards USGS 40, USGS 41 and IAEA 600. The measurement precision (1σ) was ± 0.15‰.

Samples for δ^13^C carbonate analysis were reacted with 100% H_3_PO_4_ at 70 °C to produce CO_2_. The isotope measurements were carried out using a KIEL IV device connected online to a FinniganMAT Delta plus isotope mass spectrometer (IGS PAS, Warsaw). Results are expressed in δ notation relative to the VPDB and normalized to international standards NBS 18, NBS 19 and IAEA-CO-9. The measurement precision was better than ± 0.08‰.

### Total organic carbon and total sulphur

Total carbon (TC), total inorganic carbon (TIC) and total sulphur contents were measured using an Eltra CS-500 IR-analyser with a TIC component. Calibration of the analyser was performed using the Eltra standards. Approximate carbonate content (CC), assuming that all carbonates are calcite, was calculated from the formula

CC = TIC × 8.3333.

TOC content was calculated by subtracting the TIC content the TC content.

Analytical precision and accuracy were better than ± 2% for TC and ± 3% for TIC. For details, see^9^.

### Inorganic geochemistry

Twenty-five pulverized bulk-rock samples were analysed at Bureau Veritas Acme Labs Canada Ltd. Major, minor, and trace elements were analysed using inductively coupled plasma optical emission spectrometry and inductively coupled plasma mass spectrometry. The precision and accuracy of the results were better than ± 0.05% (mostly ± 0.01%) for major elements and generally better than ± 1 ppm for trace elements.

### Mercury determination

Pulverized bulk-rock samples were analysed using a two-cell, pyrolyser-type Milestone DMA-80 Direct Mercury Analyser for atomic absorption spectrometry at the Institute of Earth Sciences, University of Silesia (Poland). Analytical details are described in^9^.

## Supplementary Information

Supplementary Information
