# Supplementary material for: Mercury spikes as evidence of extended arc-volcanism around the Devonian–Carboniferous boundary in the South Tian Shan (southern Uzbekistan)
Source: Sci Rep. 2021 Mar 11;11:5708. doi: 10.1038/s41598-021-85043-6 (PMC7970954; doi:10.1038/s41598-021-85043-6)

**SUPPLEMENTARY DATA**

**Mercury spikes as evidence of extended arc-volcanism around the Devonian-Carboniferous boundary in the South Tian Shan (southern Uzbekistan)**

Michał Rakociński, Agnieszka Pisarzowska, Carlo Corradini, Narkiewicz Katarzyna, Zofia Dubicka, Nuriddin Abdiyev

*^1^University of Silesia in Katowice, Faculty of Natural Sciences, Institute of Earth Sciences, Będzińska 60, Sosnowiec 41- 200, Poland;*

*^2^Dipartimento di Matematica e Geoscienze, Università di Trieste, via Weiss 2, 34128 Trieste, Italy*

*^3^Polish Geological Institute – National Research Institute, Rakowiecka 4, 00-975 Warsaw, Poland*

*^4^University of Warsaw, Faculty of Geology, Żwirki i Wigury 93, PL 02-089, Warszawa, Poland*

*^5^Kitab State Geological Reserve, Shakhrisabz, Kashkadarya Region, Uzbekistan*

*corresponding author: michal.rakocinski@us.edu.pl

**SD. 1.** Conodont occurrences in the Novchomok (22) section (Nov 22). Abbreviations: *Pa*. *gr*. – *Palmatolepis gracilis*; *Bi*. *cost*. – *Bispathodus costatus*; *ac*. – *aculeatus*; *Pr*. – *Protognathodus*

| Conodont zones/subzones | *Pa*. *gr*.  *manca*–*Bi*.*cost*. | *Bi. ac*. *aculeatus – Bi*. *ultimus* | | *Pr. meischneri* | ?*Pr. meischneri* | ? |
| --- | --- | --- | --- | --- | --- | --- |
| Sample numbers | 019 | 09A | 01 | 0 | 1 | 7 |
| Weight of samples (kg) | 0.25 | 0.13 | 0.25 | 0.48 | 0.24 | 0.48 |
| *Protognathodus collinsoni* |  |  |  | 3 |  |  |
| *Protognathodus* cf. *collinsoni* |  |  |  | 2 |  |  |
| *Protognathodus collinsoni→kockeli* |  |  |  | 3 |  |  |
| *Protognathodus* sp. |  |  |  | 3 |  |  |
| *Pseudopolygnathus* sp. |  |  |  | 1 |  | 1 |
| *Palmatolepis gracilis sigmoidalis* |  |  |  | 3 |  |  |
| *Palmatolepis gracilis* |  |  |  | 2 |  |  |
| *Palmatolepis perlobata* |  |  |  | 3 |  |  |
| *Palmatolepis* sp. |  | 1 |  | 1 | 1 |  |
| *Bispathodus* cf.*B.*  *aculeatus aculeatus* |  | 1 |  |  |  |  |
| *Polygnathus vogesi* |  |  | 1 |  |  |  |
| *Polygnathus* aff. *P*. *styriacus* |  | 1 |  |  |  |  |
| *Polygnathus communis renatae* |  |  |  | 3 |  |  |
| *Polygnathus communis*  *dentatus* | 1 |  |  | 1 |  |  |
| *Polygnathus communis* aff. *dentatus* |  |  |  | 1 |  |  |
| *Polygnathus communis communis* | 2 |  |  | 10 |  |  |
| *Polygnathus communis* ssp. A |  |  |  | 2 |  |  |
| *Polygnathus communis* ssp. B |  |  |  | 2 |  |  |
| *Polygnathus communis* |  |  |  | 14 |  |  |
| *Polygnathus* sp. | 1 | 1 | 5 | 45 | 4 |  |
| *Pseudopolygnathus controversus* M 1 | 1 |  |  |  |  |  |
| *Pseudopolygnathus* cf. *brevipennatus* |  |  |  | 1 |  |  |
| *Pseudopolygnathus* aff. *Ps*. *primus* | 1 |  |  | 1? |  |  |
| *Pseudopolygnathus* sp. | 2 |  |  |  |  |  |
| *Belodella* sp. |  |  |  | 1 |  |  |
| *Panderodus* sp. |  |  |  | 1 |  |  |
| Ramiforms | 0 | 4 |  |  |  |  |
|  | 8 | 4 | 6 | 101 | 5 | 1 |

**SD. 2.** Selected characteristic conodont elements from the Novchomok-22 section


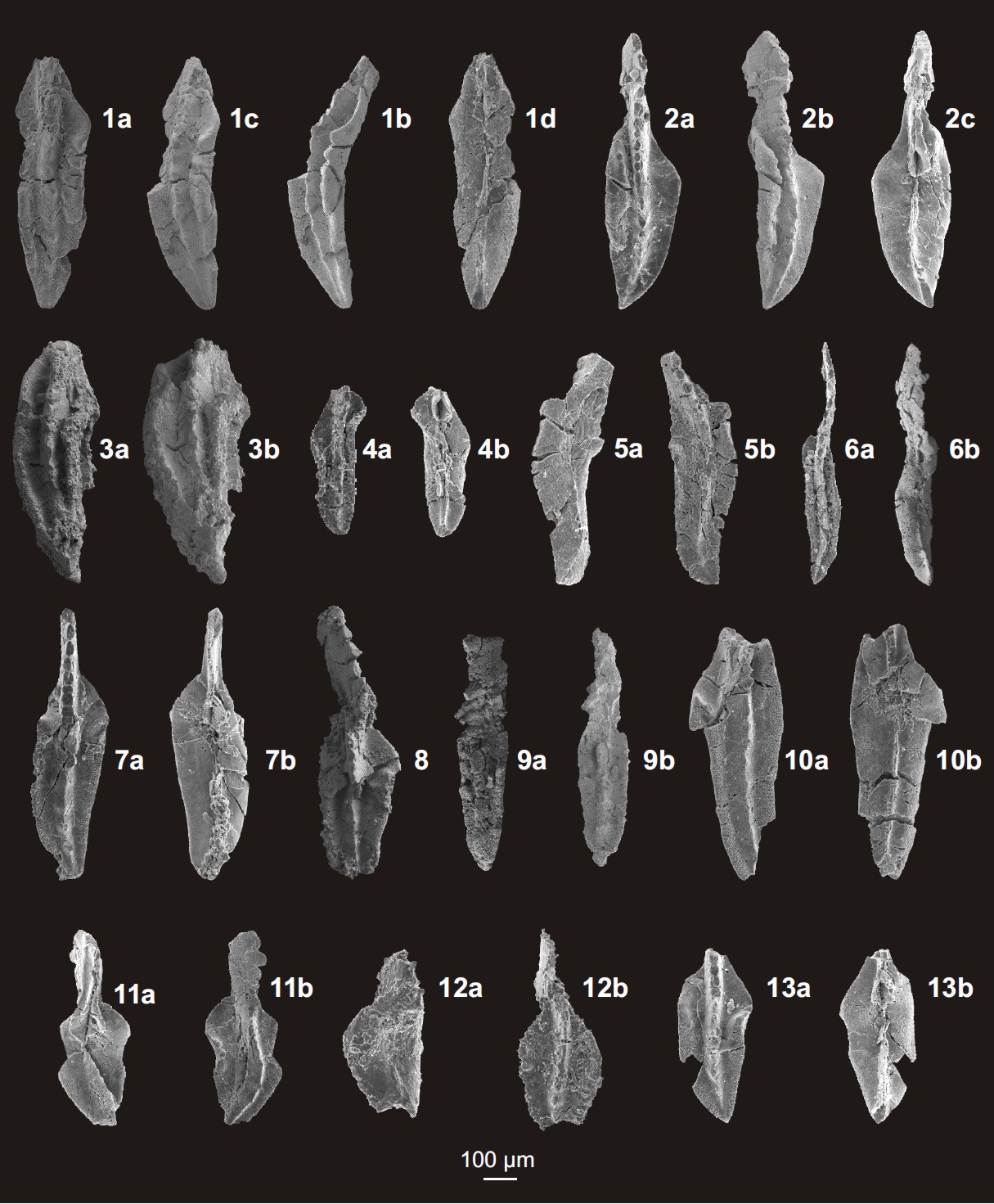


All conodont elements (P_1_) are from the sample Nov. 22-0.

1. *Polygnathus communis dentatus* Druce (1a. upper, 1b. oblique, 1c. lateral, 1d. lower views, MUZ PIG 1825.II.24), 2. *Polygnathus communis* aff. *dentatus* Druce (2a. upper, 2b. lateral/oblique, 2c. lower views, MUZ PIG 1825.II.25), 3, 4. *Polygnathus communis* ssp. A. (3a. upper/oblique, 3b. upper views, MUZ PIG 1825.II.26; 4a. upper, 4b. lower views of juvenile form, MUZ PIG 1825.II.27), 5, 6. *Polygnathus communis* ssp. B. (5a. lower/oblique, 5b. upper views, MUZ PIG 1825.II.28; 6a. upper, 6b. lower views of juvenile form, MUZ PIG 1825.II.29), 7, 8. *Polygnathus communis renatae* Corradini and Spalletta (7a. upper, 7b. lower views, MUZ PIG 1825.II.30; 8. upper view, MUZ PIG 1825.II.31), 9, 10. *Polygnathus* sp. (9a. oblique, 9b. upper views of juvenile form, MUZ PIG 1825.II.32; 10a. upper, 10b. lower views, MUZ PIG 1825.II.33), 11-13. *Polygnathus communis communis* Branson and Mehl (11a. lower, 11b. upper views, MUZ PIG 1825.II.34; 12a. lower, 12b. upper views, MUZ PIG 1825.II.35; 13a. upper, 13b. lower views, MUZ PIG 1825.II.36).

Illustrated specimens were attributed to *P*. *communis* ssp. A (SD2: 3, 4) based on the observation that they possess small elongated basal pit with thickened margins protruding above the platform surface in its anterior part. Behind the basal pit there is a depression characteristic for *Polygnathus communis*. Behind the depression a narrow elevated keel is visible. Platform is smooth, narrow, elongated, with a pointed posterior top. Carina is fairly massive and consisting of densely arranged nodes which are smaller and more widely spaced posteriorly. Carina can be straight or slightly curve and its height is more or less equal to upturned platform margins which run parallel to the carina. The outer margin is shorter than the inner one and therefore the geniculation points are not opposite. Behind the geniculation points the anterior trough margins join the blade at the same level. The outer anterior trough is longer and it is widest just behind the geniculation point, and its margin runs obliquely to the blade. The inner one is much shorter and its margin is rounded.

The illustrated specimens *Polygnathus communis* ssp. B. (SD2: 5, 6) are very similar to the representatives of *P*. *communis* ssp. A, they differ in possessing markedly sigmoidal blade and carina, similarly as platform margins running parallel to the carina.

**SD. 3.** (A) δ^13^C_org_ vs Hg, (B) δ^13^C_org_ vs TOC, (C) δ^13^C_carb_ vs Hg, and Hg vs TOC scatter diagrams for the Novchomok section. Increases in Hg content correlate with lower carbon isotope values. Note that high Hg concentrations do not correlate with increases in TOC contents.


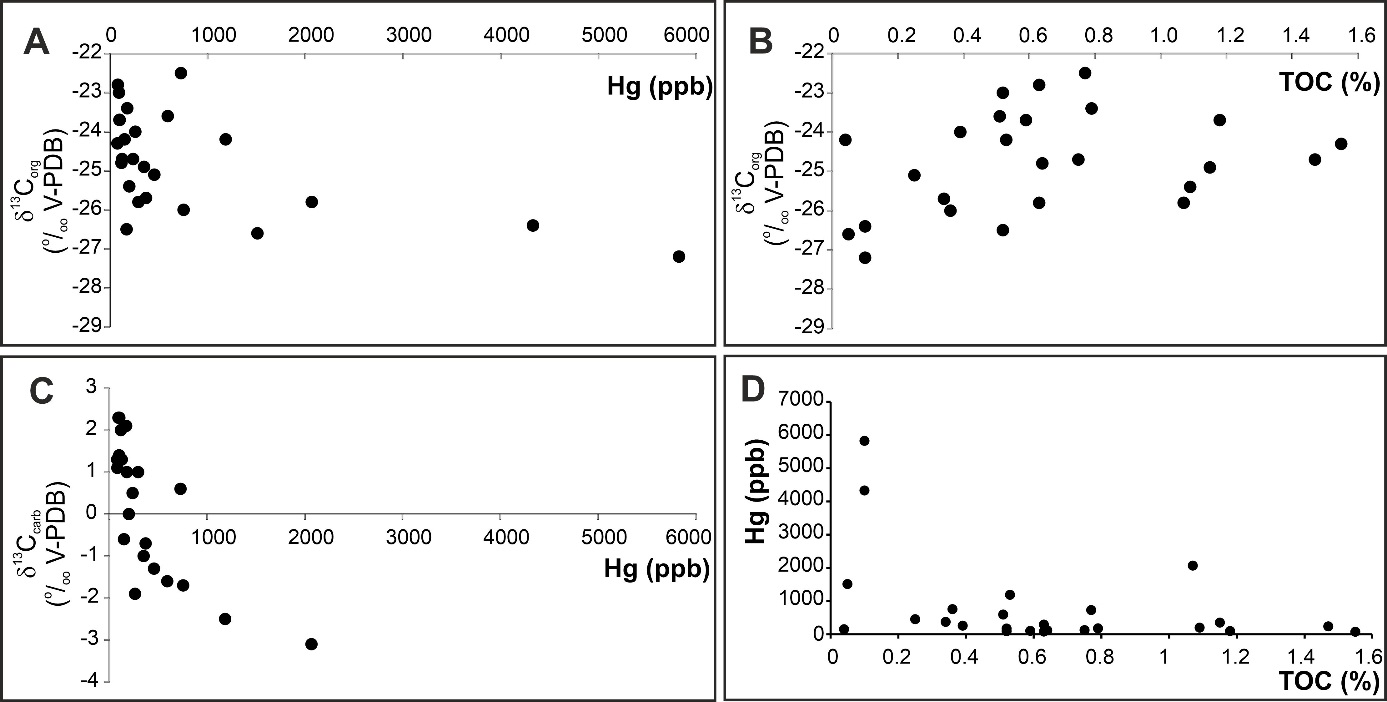

Supplement: Supplementary file 1 — Supplementary Information [file 41598_2021_85043_MOESM1_ESM.docx]
